# Effect of dietary tall oil fatty acids and hydrolysed yeast in SNP2-positive and SNP2-negative piglets challenged with F4 enterotoxigenic *Escherichia coli*

**DOI:** 10.1038/s41598-024-52586-3

**Published:** 2024-01-24

**Authors:** Anouschka Middelkoop, Hannele Kettunen, Xiaonan Guan, Juhani Vuorenmaa, Ramon Tichelaar, Michela Gambino, Martin Peter Rydal, Francesc Molist

**Affiliations:** 1 Schothorst Feed Research B.V., 8218 NA Lelystad, The Netherlands; 2 Hankkija Oy, 05800 Hyvinkää, Finland; 3 Department of Veterinary and Animal Sciences, Faculty of Health and Medical Sciences, University of Copenhagen, 1870 Frederiksberg C, Denmark

**Keywords:** Animal breeding, Pathogens

## Abstract

Reduction of post-weaning diarrhoea caused by ETEC is a principal objective in pig farming in terms of welfare benefits. This study determined the effects of genetic susceptibility and dietary strategies targeting inflammation and fimbriae adherence on F4-ETEC shedding and diarrhoea in weaned piglets in an experimental challenge model. A DNA marker test targeting single nucleotide polymorphism 2 (SNP2) identified piglets as heterozygous (SNP2+, susceptible) or homozygous (SNP2-, resistant) to developing F4ac-ETEC diarrhoea. A total of 50 piglets, 25 SNP2+ and 25 SNP2-, were weaned at 30 days of age and equally distributed to different treatments (n = 10): Positive control (PC): piglets fed with a negative control diet and provided with colistin via drinking water; Negative control (NC): piglets fed with a negative control diet; Tall oil fatty acids (TOFA): piglets fed with a negative control diet + 1.0 g TOFA/kg feed; Yeast hydrolysate (YH): piglets fed with a negative control diet + 1.5 g YH/kg feed derived from *Saccharomyces cerevisiae*; and Combination (COM): piglets fed with a negative control diet + 1.0 g TOFA and 1.5 g YH/kg feed. On day 10 post-weaning, all piglets were infected with F4-ETEC by oral administration. Piglets fed with PC, TOFA, YH or COM had a lower faecal shedding of F4-ETEC than NC piglets (*P* < 0.001), which was also shorter in duration for PC and TOFA piglets than for NC piglets (*P* < 0.001). Piglets in PC, TOFA, YH and COM had a shorter diarrhoea duration versus NC when classified as SNP2+ (*P* = 0.02). Furthermore, PC, TOFA and YH piglets grew more than NC and COM piglets in the initial post-inoculation period (*P* < 0.001). In addition, the level of faecal F4-ETEC shedding and the percentage of pigs that developed F4-ETEC diarrhoea (72 vs. 32%, *P* < 0.01) following infection were higher, and the duration of F4-ETEC diarrhoea longer (2.6 vs. 0.6 days, *P* < 0.001), in SNP2+ piglets than in SNP2- piglets, and led to reduced growth performance (*P* = 0.03). In conclusion, piglets fed with TOFA, YH or their combination, irrespective of their SNP2 status, are more resilient to F4-ETEC infection. Moreover, SNP2+ piglets show a higher level of F4-ETEC shedding and diarrhoea prevalence than SNP2- piglets, confirming an association between SNP2 and F4ac-ETEC susceptibility.

## Introduction

In pig production, post-weaning diarrhoea (PWD) is a common gastrointestinal problem for newly weaned piglets, characterized by watery faeces, dehydration, and reduced appetite and growth, and it can even lead to death^1^. Enterotoxigenic *E. coli* (ETEC) is the most common pathogen associated with PWD^2^.

Reducing PWD caused by ETEC and the associated antimicrobial usage is crucial to improve piglet health and welfare and to restrict the spread of antimicrobial resistance among ETEC^1,3,4^. Since therapeutic levels of zinc oxide cannot be used anymore for treating PWD within the European Union due to environmental and antimicrobial resistance concerns^5^, functional feed additives, feed ingredients and feeding strategies have become fundamental in reducing antimicrobials in pig production to sustain the health status and reduce the risk of pathologies. Although different in-feed additives and dietary formulations have been recognized as promising dietary alternatives^6,7^, to date no single ‘silver bullet’ has been found that can replace antibiotics and zinc oxide. Additionally, the implementation of genotype findings in breeding programs is important for reducing ETEC PWD. An integrated approach to control PWD should be considered, including the impact of nutrition, genetics, housing and management. In the current study, we investigated the effect of two distinct classes of registered feed material in the European Union on PWD in piglets of diverse genetic backgrounds.

Tall oil fatty acid (TOFA) is a standardized composition of free fatty acids and coniferous resin acids (see Supplementary Table 1, Additional File 1). The resin acids of TOFA are lipophilic diterpene carboxylic acids with antimicrobial, antifungal and anti-inflammatory activities^8–11^. In previous in vivo studies, dietary TOFA was shown to improve performance and reduce PWD^12^ and beneficially affect the immunological status of piglets^13^. Yeast and yeast derivatives are often used in farm animal diets because of their nutritional value and their positive effects on animal health and performance^14–16^. A *Saccharomyces cerevisiae*-based yeast hydrolysate (YH) has been shown to reduce PWD^17^, and to improve the growth performance, feed conversion and immune response of piglets^18^. However, it has not yet been fully investigated whether TOFA and YH supplementation can prevent F4-ETEC associated PWD development.

To enable colonisation and proliferation, the fimbriae of ETEC adhere to specific host receptors on the brush border of enterocytes in the small intestine, followed by endotoxin production that causes diarrhoea^19^. The most prevalent antigenic variant of ETEC associated with PWD in piglets is F4ac-ETEC^20,21^. Previously, MUC4 and MUC13 were suggested to be the gene responsible for the intestinal receptor that allows ETEC to adhere to the intestinal tract^22,23^; however, later studies rejected this hypothesis and proposed a refined candidate region for F4ac ETEC on chromosome 13^24^. Goetstouwers et al.^24^ reported two significant and completely linked SNPs (SNP1 and SNP2) on chromosome 13 close to MUC13 that were found to be strongly associated with F4ab/ac receptor phenotyping based on in vitro F4ac-ETEC adherence to intestinal cells, suggesting that further investigation is needed into these SNPs as a marker for in vivo F4ac-ETEC susceptibility.

The aim of this study was to evaluate the protective effect of dietary inclusion of TOFA and YH against F4ac-ETEC infection in the post-weaning period, and to evaluate the possible additional benefits of combining both dietary strategies. It was hypothesized that dietary supplement TOFA, YH and their combination could prevent or limit the detrimental effects of ETEC infection improving animal health and performance. Moreover, these strategies were evaluated in SNP2-positive (SNP2 +) and SNP2-negative (SNP2-) piglets to provide more information regarding the potential role of SNP2 as a genetic susceptibility marker in a pilot study setup. It was hypothesized that SNP2+ piglets would exhibit a higher shedding of F4-ETEC, a higher prevalence of PWD, and reduced growth performance compared to SNP2- piglets.

## Results

### Dietary tall oil fatty acids and hydrolysed yeast as well as genetics linked to F4-ETEC resistance reduce F4-ETEC PWD severity and duration

Faecal dry matter analyses were in line with the faecal scoring system to classify faeces into diarrhoeic (score ≤ 4) and non-diarrhoeic samples (score > 4). The faecal dry matter percentages were; 9.1 ± 3.0% for score 2 (n = 2), 13.7 ± 3.8% for score 3 (n = 20), 18.2 ± 7.3% for score 4 (n = 13), 23.3 ± 3.7% for score 5 (n = 5), 31.8 ± 5.2% for score 6 (n = 8), 37.4 ± 6.0% for score 7 (n = 9), 43.6 ± 4.1% for score 8 (n = 7) and 43.0 ± 2.5% for score 9 (n = 5).

Inoculation with the ETEC strain resulted in a drop in faecal score to 5.1 on day 12 post-weaning, i.e., two days post-inoculation (day 2 PI), and recovered thereafter until an average score of 5.8 on day 22 post-weaning, i.e., day 12 PI (see Supplementary Fig. 1, Additional File 1).

**Figure 1:**
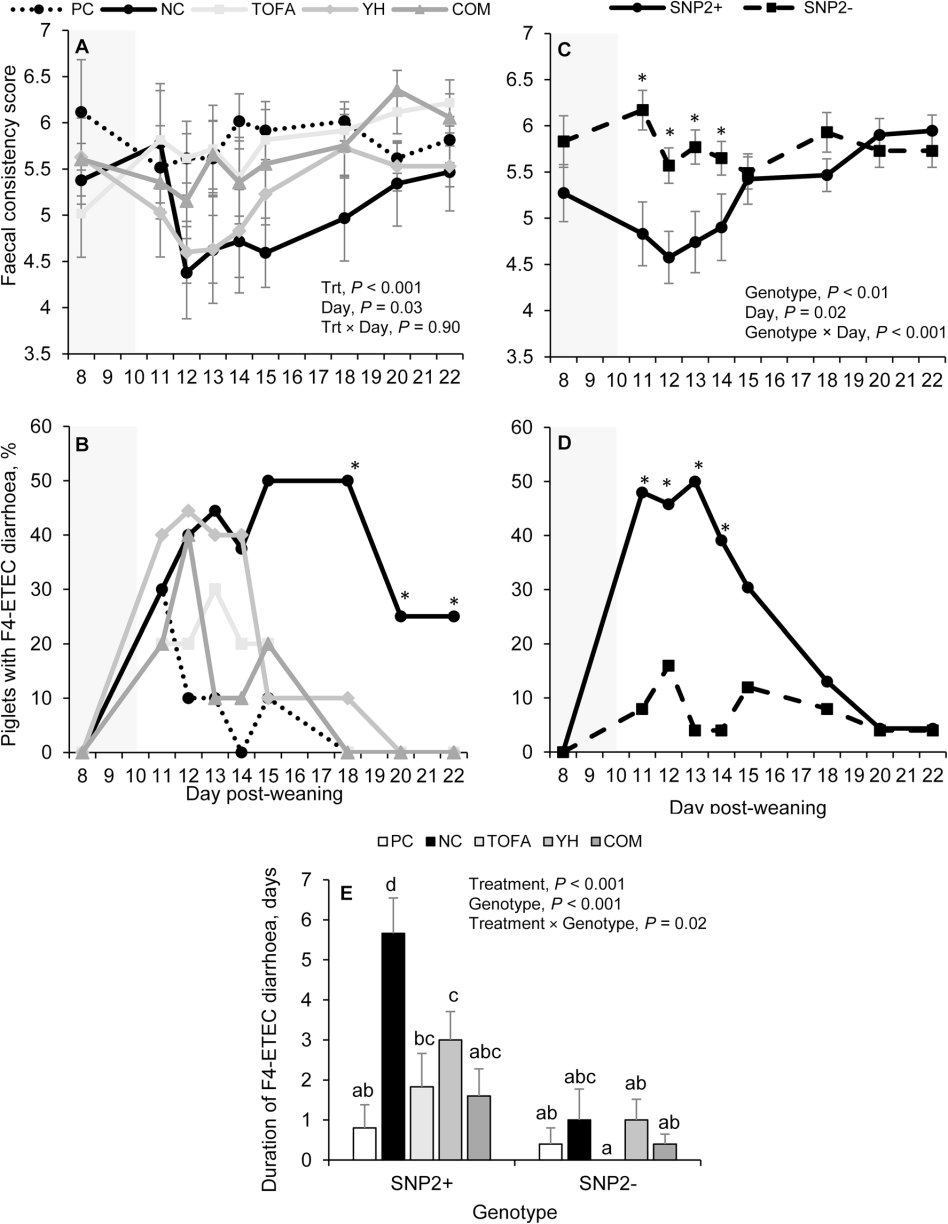
Faecal consistency (**A**, **C**), prevalence of F4-ETEC diarrhoea (**B**, **D**), and the duration of F4-ETEC diarrhoea (**E**). F4-ETEC inoculation took place on day 10 post-weaning in piglets of diverse genetic backgrounds fed with different dietary strategies. The grey-coloured background indicates the pre-inoculation period. PC = control diet + colistin via drinking water; NC = control diet; TOFA = 1.0 g tall oil fatty acids per kg feed; YH = 1.5 g yeast hydrolysate from *Saccharomyces cerevisiae* per kg feed; COM = 1.0 g TOFA and 1.5 g YH per kg feed; Trt = treatment.

The effect of treatment and genotype on the pattern of faecal consistency and diarrhoea is summarized in Fig. 1. No interaction between treatment and day was found on faecal consistency (Fig. 1A), but treatment affected it significantly (*P* < 0.001); an improved faecal consistency score was found for PC (5.8 ± 0.11^b^), TOFA (5.7 ± 0.13^b^) and COM piglets (5.6 ± 0.12^b^) versus NC (5.0 ± 0.17^a^) and YH piglets (5.2 ± 0.13^a^). From day 18 to 22 post-weaning the proportion of piglets with F4-ETEC PWD was significantly higher in the NC group than in the other groups (Fig. 1B). In total, 30% of PC piglets, 70% of NC piglets, 30% of TOFA piglets, 70% of YH piglets, and 60% of COM piglets developed F4-ETEC PWD after inoculation (*P* = 0.16).

Genotype and day did interact (*P* = 0.008), showing that SNP2+ piglets had a lower faecal consistency score than SNP2- piglets during the first four days post-inoculation, i.e., from day 11 to 14 post-weaning (Fig. 1C).

Moreover, a significant difference in the prevalence of F4-ETEC PWD was observed between SNP2+ and SNP2- piglets, in which 72% of the SNP2+ versus 32% of the SNP2- piglets developed F4-ETEC PWD (*P* < 0.01). This difference in F4-ETEC PWD prevalence between SNP2+ and SNP2- piglets was particularly present during the first four days post-inoculation (Fig. 1D).

The duration of F4-ETEC PWD was significantly affected by the interaction between treatment and genotype (Fig. 1E). PC, TOFA, YH and COM resulted in a shorter duration of diarrhoea than NC in SNP2+ piglets. However, this effect was not observed in SNP2- piglets, which showed a similar and short duration of diarrhoea across all treatment groups. Looking for the main effects, PC (0.6 ± 0.34^a^ days), TOFA (1.1 ± 0.57^a^ days), YH (1.8 ± 0.51^a^ days) and COM piglets (1.0 ± 0.39^a^ days) had a shorter duration of F4-ETEC PWD than NC piglets (3.3 ± 1.01^b^ days, *P* < 0.001), and SNP2+ piglets had a longer duration of diarrhoea than SNP2- piglets (2.6 ± 0.44 vs. 0.6 ± 0.22 days, *P* < 0.001).

### Dietary tall oil fatty acids and hydrolysed yeast reduce F4-ETEC shedding, while piglets of SNP2+ genotype shed higher quantities of F4-ETEC than piglets of SNP2- genotype

No F4-ETEC was detected in faeces before the challenge, and all piglets had quantifiable amounts of F4-ETEC on the first day after the challenge. F4-ETEC shedding was affected by day with the highest F4-ETEC shedding on the first day after inoculation (day 1 PI), which decreased on day 2 PI and remained constant until day 5 PI, after which it decreased further on days 8 and 10 PI (see Supplementary Fig. 2, Additional File 1).

**Figure 2:**
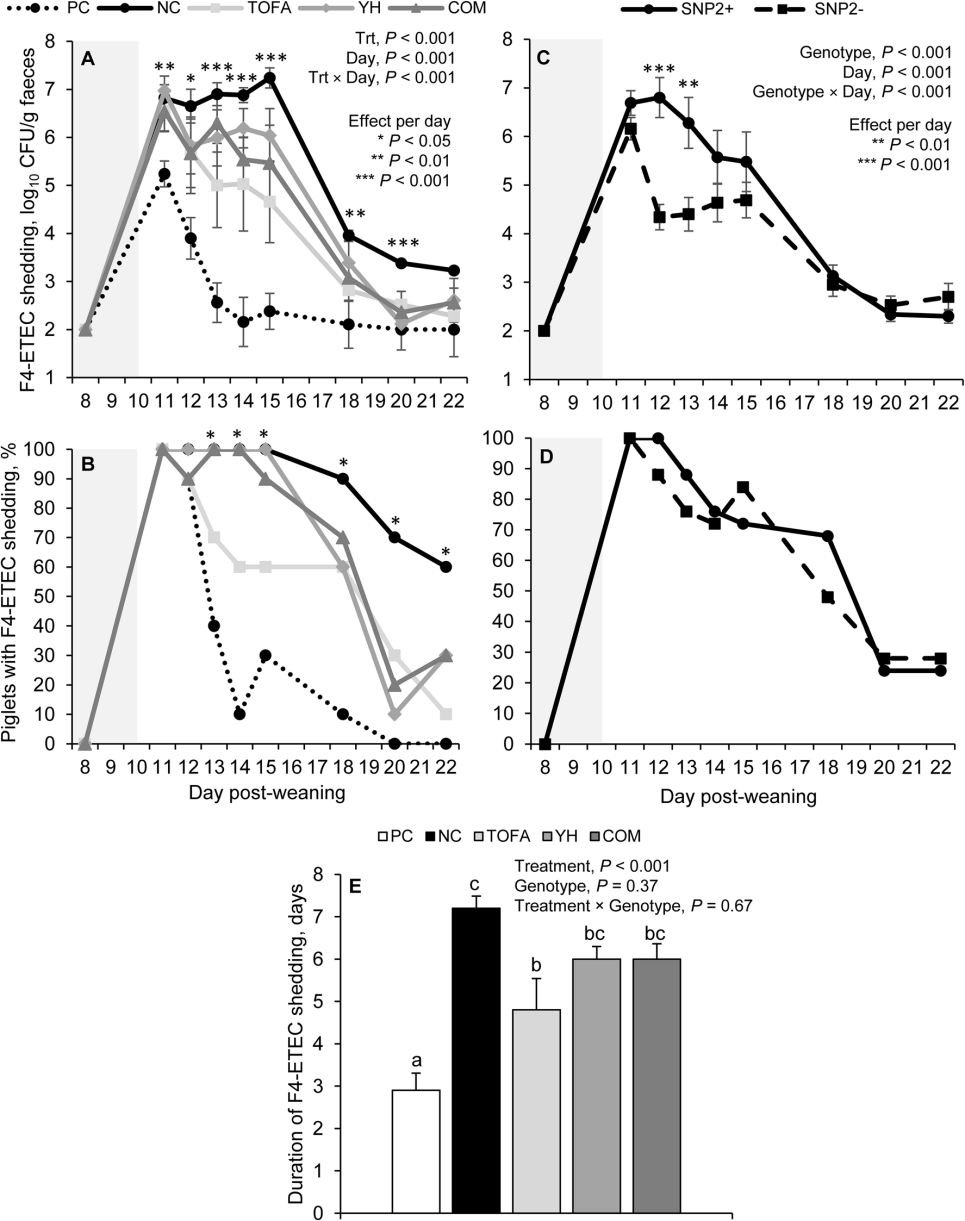
Quantitative faecal F4-ETEC shedding (**A**, **C**), prevalence of F4-ETEC shedding (**B**, **D**) and the duration of F4-ETEC shedding (**E**). F4-ETEC inoculation took place on day 10 post-weaning in piglets of diverse genetic backgrounds fed with different dietary strategies. The grey-coloured background indicates the pre-inoculation period. PC = control diet + colistin via drinking water; NC = control diet; TOFA = 1.0 g tall oil fatty acids per kg feed; YH = 1.5 g yeast hydrolysate from *Saccharomyces cerevisiae* per kg feed; COM = 1.0 g TOFA and 1.5 g YH per kg feed; Trt = treatment.

Faecal F4-ETEC shedding was significantly affected by treatment (Fig. 2A) and genotype (Fig. 2C), but not by their interaction (*P* > 0.10, see Supplementary Table 6, Additional File 1). Treatment did significantly interact with day for faecal F4-ETEC shedding (*P* < 0.001), showing that treatment affected faecal F4-ETEC shedding at all measurement days, except for days 8 and 22 post-weaning (Fig. 2A). As expected, piglets in the PC group shed consistently less faecal F4-ETEC than piglets in the NC group from day 11 to 20 post-weaning and than piglets fed with TOFA, YH and COM from day 11 to 15 post-weaning. Piglets fed TOFA excreted less faecal F4-ETEC than piglets fed with the NC diet from day 13 to 20 post-weaning. On day 18 post-weaning the shedding of F4-ETEC in piglets fed with TOFA did not differ from that of piglets in the PC group. On day 20 post-weaning, NC-fed piglets had more F4-ETEC in their faeces than piglets in the PC, TOFA, YH and COM groups. Altogether, F4-ETEC shedding was affected by treatment (*P* < 0.001), with piglets in PC (2.71 ± 0.128^a^) showing significantly less F4-ETEC shedding than piglets fed with TOFA (4.06 ± 0.262^b^), YH (4.57 ± 0.244^c^) and COM (4.39 ± 0.240^bc^), which all shed significantly less faecal F4-ETEC than piglets fed NC (5.22 ± 0.252^d^ log_10_ CFU/g faeces). Piglets fed TOFA also had lower levels than piglets fed YH. From days 13 to 22 post-weaning, the prevalence of faecal F4-ETEC shedding was significantly affected by treatment (Fig. 2B). The highest prevalence was found for piglets fed with NC, the lowest prevalence of piglets in PC and intermediate prevalence for TOFA (day 13 to 22), YH (day 18 to 22) and COM (day 18 to 22), with TOFA having the closest effect to PC. Moreover, PC and TOFA piglets shed F4-ETEC for fewer days than NC piglets (Fig. 2E).

SNP2+ piglets shed significantly higher faecal F4-ETEC concentrations at days 12 and 13 post-weaning compared to SNP2- piglets (genotype × day interaction, Fig. 2C). The main effect of genotype showed that SNP2+ piglets had more faecal F4-ETEC shedding than SNP2- piglets (4.51 ± 0.180 vs. 3.82 ± 0.125 log_10_ CFU/g faeces, *P* < 0.001). The prevalence of F4-ETEC shedding tended to be higher in SNP2+ (100% of the piglets shedding F4-ETEC in the faeces) than SNP2- piglets on day 12 post-weaning (88%, *P* = 0.08), but not at the other days (*P* ≥ 0.10, Fig. 2D). Additionally, SNP2+ piglets did not differ from SNP2- piglets in the duration of F4-ETEC shedding (5.57 ± 0.370 vs. 5.19 ± 0.437 days, *P* = 0.37) and no interaction between treatment and genotype was found for the duration of F4-ETEC shedding (*P* = 0.67; see Supplementary Fig. 3, Additional File 1).

### Dietary tall oil fatty acids or hydrolysed yeast and a homozygous resistant SNP2 genotype reduce the risk of mortality and growth retardation following F4-ETEC inoculation

Mortality due to a humane endpoint tended to be affected by treatment (*P* = 0.09). Two SNP2+ piglets from the NC group reached a humane endpoint, one on day 2 PI and one on day 3 PI. Pathology results confirmed an intestinal disorder due to ETEC infection without other abnormalities. No mortality occurred in the other treatment groups. Piglet BW and ADG were significantly affected by treatment and genotype, but not by their interaction (Table 1; Supplementary Table 7, Additional File 1). Piglet ADG from day 8 to 15 post-weaning showed significant differences between treatments (*P* < 0.001), with a better growth performance for piglets in the PC, TOFA and YH treatments versus the NC and COM treatments. SNP2+ piglets grew less than SNP2- piglets from day 8 to 15 post-weaning (*P* = 0.002) as well as overall from day 0 to 22 post-weaning (*P* = 0.03). This resulted in a significantly lower BW on day 15 post-weaning (*P* = 0.02) as well as a trend for a lower BW on day 22 post-weaning (*P* = 0.07).

**Table 1:** The effect of treatment and genotype on the performance of weaned piglets challenged with F4-ETEC^1^.

	Treatment^2^					SEM	Genotype		SEM	*P*-value		
	PC	NC	TOFA	YH	COM		SNP2+	SNP2-		T^3^	G^4^	T × G^5^
Body weight, kg												
D 0	7.88	7.80	7.84	7.79	7.78	0.356	7.85	7.78	0.227	1.00	0.83	0.90
D 8	8.55	9.08	9.06	8.98	9.22	0.408	8.87	9.09	0.260	0.82	0.57	0.57
D 15	11.72	10.55	11.71	11.69	11.13	0.442	10.84	11.88	0.282	0.27	0.02	0.35
D 22	15.59	14.02	15.71	15.27	14.68	0.608	14.53	15.58	0.338	0.28	0.07	0.64
Average daily gain, g/piglet												
D 0–8	84	160	153	149	180	27.1	127	163	17.3	0.15	0.16	0.32
D 8–15	452^b^	261^a^	379^b^	387^b^	273^a^	32.0	302	399	20.4	< 0.001	< 0.01	0.17
D 15–22	554	496	571	511	507	38.8	527	529	24.7	0.59	0.95	1.00
D 0–22	351	293	358	340	314	22.1	308	354	14.1	0.23	0.03	0.57

Different superscripts (^ab^) within a row indicate a significant difference between treatments (*P* < 0.05).

^1^F4ac-ETEC challenge on day 10 post-weaning.

^2^PC = control diet + colistin via drinking water; NC = control diet; TOFA = 1.0 g tall oil fatty acids per kg feed; YH = 1.5 g yeast hydrolysate from *Saccharomyces cerevisiae* per kg feed; COM = 1.0 g TOFA and 1.5 g YH per kg feed.

^3^ T = Treatment effect.

^4^G = genotype effect.

^5^ T x G = interaction between treatment and genotype.

## Discussion

The main response parameters in the F4-ETEC challenge model are faecal F4-ETEC shedding (prevalence, duration and quantitative level) and F4-ETEC PWD (prevalence, duration and faecal consistency score). In the present study, F4-ETEC inoculation induced F4-ETEC shedding in the faeces of all piglets on the first day after the challenge and induced clinical signs of diarrhoea with a peak post-challenge. Firstly, to compare the results of the two control groups; piglets in PC had a significantly lower prevalence, duration and level of faecal F4-ETEC shedding, as well as a lower prevalence and duration of PWD, than piglets in NC. Temporarily reduced piglet performance could be observed, as PC piglets had better feed efficiency (1.02 ± 0.032 vs. 1.37 ± 0.001) and growth performance than NC piglets during the initial post-inoculation period, i.e., from day 8 to 15 post-weaning. These results confirmed effective inoculation. It should be noted that colistin was provided in the drinking water of PC piglets throughout the 22 days of the trial. This differs from the practical application of colistin, which should be limited to the prescription period. The farm animal industry aims to minimize the use of antimicrobials such as colistin, to reduce antimicrobial resistance^25^, highlighting the importance of reducing F4-ETEC infections by breeding, housing, management and nutritional strategies.

This trial aimed to expand our knowledge in the search for non-antibiotic alternatives for managing PWD. To that aim, the effect of dietary supplementation based on tall oil fatty acids or a yeast hydrolysate and the impact of adding both feed ingredients was assessed in F4-ETEC-challenged piglets of diverse genetic backgrounds. Genotype (SNP2+ or SNP2-) as well as the two nutritional strategies (TOFA and YH), either applied alone in the diet or in combination (COM), significantly affected the response of weaned piglets to a F4ac-ETEC challenge. Supplementation of weaned piglets with either 0.1% dietary TOFA or 0.15% dietary YH resulted in a lower prevalence of F4-ETEC detected in faecal samples, less faecal shedding of F4-ETEC, a shorter duration of PWD and improved growth performance in the first days post-inoculation compared to a non-supplemented diet. TOFA also shed faecal F4-ETEC for a shorter number of days and exhibited an improved faecal consistency. These results are in line with the in vitro efficacy of TOFA in an *E. coli* O149 assay (Roy et al., 2018), a reduction in PWD and improved growth at 3 weeks post-weaning observed in piglets fed 0.1% TOFA in a Finnish commercial weaner facility^12^ and a reduction in ETEC shedding and PWD of weaned piglets fed with 0.3% YH from 2 weeks prior to weaning onwards^17^. In addition, the current study shows that dietary YH supplementation is also successful in reducing F4-ETEC infections of weaned piglets when applied from weaning at an inclusion rate of 0.15%. The combination of TOFA and YH resulted in a lower prevalence of faecal F4-ETEC, less faecal shedding of F4-ETEC, a better faecal consistency and a shorter diarrhoea period compared to a non-supplemented diet, but did not exert additional beneficial effects compared to the singular application of either TOFA or YH in the current trial setup. In the present study, the animal performance parameters were not considered the main response parameters, but they still provide valuable data on the severity of the disease induced by the challenge and on the effects of the dietary treatments. COM piglets numerically had the best FCR (data not shown) and ADG in the first week before the challenge when feed intake levels among treatments were similar (ranging from 196 to 228 g/piglet). While TOFA and YH improved the growth performance of piglets versus NC in the initial post-challenge period, this effect was not observed in COM. The feed consumption level of COM piglets may have played a role in this, since a numerically lower feed intake was observed in COM piglets (528 g/piglet) versus TOFA (627 g/piglet) and YH piglets (587 g/piglet) from day 8 to 22 post-weaning, which suggests that less TOFA and YH ended up in the intestines of COM piglets. The faecal consistency of piglets fed with TOFA, YH and COM and the duration of diarrhoea were similar to PC piglets, as well as the ADG between PC, TOFA and YH in the initial post-challenge period. These results emphasize the size effect of the two feed ingredients and suggest that they can partly represent an alternative to the use of antibiotics for managing PWD and sustaining piglet performance.

Investigation of the mechanism of action was beyond the scope of this trial, but in-depth analyses of biological samples in research incorporating singular and combined use of the two feed ingredients are recommended to provide further indications regarding the observed effects. The efficacy of TOFA in reducing F4-ETEC PWD may be derived from its antimicrobial^11,26^, anti-inflammatory^27^, immunomodulatory^13,28^ and gut microbiome stabilizing properties^28,29^. The TOFA product used consisted of resin acids such as abietic, dehydroabietic and pimaric acids, as well as free fatty acids such as (conjugated) linoleic, oleic and pinolenic acids. TOFA and/or its components have been shown to increase systemic IgG concentrations^28,30^ and modulate cytokine production^13,31,32^, which may limit the inflammatory response following F4-ETEC infection. Moreover, diet-derived coniferous resin acids have been shown to decrease inflammatory T-cell infiltration and inflammation-associated collagen degradation in the intestinal tissue of broiler chickens^27^, and it is possible that they may have the same effect in piglets, thereby improving intestinal integrity and reducing PWD. In addition, TOFA might disrupt the bacterial cell wall and membrane of pathogens^26^, which may have lowered the abundance of F4-ETEC in the intestine, and may modulate the microbiota population in such a way that piglets are less susceptible to ETEC infection, for example via the production of anti-inflammatory and energy-providing metabolites^12,33^. The latter may also play a role in the improved performance as a result of TOFA feeding that has been observed in challenged^13,34^ and non-challenged animals^12,35,36^.

The tested YH was an acid-hydrolysed spent *Saccharomyces cerevisiae* brewer's yeast, which contained both soluble and insoluble yeast cell wall-derived components, including mannans, oligosaccharides, peptides and nucleotides. The mannan oligosaccharides (MOS) in YH may have prevented attachment of F4-ETEC to the intestinal epithelial cells by adhesion to the mannose-binding proteins that are expressed on the fimbriae of ETEC strains^37,38^. YH may also protect weaned piglets from F4-ETEC challenge by a higher innate and adaptive immune response, including higher (natural) antibody production, as seen in weaned piglets challenged with *E. coli* lipopolysaccharides^39^ and sheep red blood cells^18^, and in calves exposed to a vaccine challenge^40^. β-glucans from the yeast cell wall were found to attenuate intestinal damage upon F4-ETEC challenge in weaned piglets via an improved intestinal morphology, such as increased villus length and increased expression levels of tight junction proteins, suppression of inflammatory cytokines, and changes in the microbial population and fermentation pattern^41^. In addition, nucleotide rich yeast extract was reported to reduce PWD caused by ETEC^42^, and was suggested to play a role as a gut microbiome stabilizer^43–45^ and immune-stimulant^46^. YH is also known to improve nutrient digestibility and feed efficiency, resulting in improved body weight gain in pigs^47–49^.

Due to the number of piglets, this study has limited experimental power for the interaction between treatment and genotype, particularly for the response parameter piglet performance. This may explain the lack of an interaction effect between treatment and genotype on piglet ADG and BW, while such an interaction effect was reported for the duration of F4-ETEC diarrhoea. The lack of an interaction effect of treatment and genotype in combination with the significant treatment effect on the level and duration of faecal F4-ETEC shedding suggests that PC, TOFA, YH and COM lowered the level and shortened the duration of faecal F4-ETEC shedding compared to NC, not only in SNP2+ but also in SNP2- piglets. It therefore seems highly relevant to implement such nutritional strategies on pig farms, in which a distribution of F4-susceptible and F4-resistant animals can also be found^50^ and in which less F4-ETEC shedding is considered important for low infection pressure and to prevent secondary and/or coinfections, such as by *Streptococcus suis*^51^.

As hypothesized from the results of ^24^, SNP2+ piglets had a higher prevalence and shedding of faecal F4-ETEC, as well as a higher prevalence and longer duration of F4-ETEC diarrhoea than SNP2- piglets. SNP2+ piglets also had a lower growth rate than SNP2- piglets, resulting in a lower body weight of the piglets. These observations were mainly present within the first 5 days PI, suggesting that SNP2 is associated with the susceptibility of piglets to F4ac-ETEC. These results imply that it is important to select and allocate pigs to experimental treatments based on their F4ac-susceptibility profile to eliminate this confounding factor in nutritional studies using the ETEC-F4 infection model. Moreover, the results indicate that SNP2 is a promising marker for screening experimental animals to determine F4ac susceptibility, and support the results of ^24,52^. To the best of our knowledge, this is the first pilot study that compared SNP2+ with SNP2– piglets in an ETEC challenge study in weaned piglets. Of the SNP2+ piglets, 72% developed F4-ETEC PWD. The remaining 28% of the SNP2+ piglets did not develop diarrhoea, of which 12% received colistin as part of PC, 12% were fed with TOFA and 4% were fed with COM, which are the three interventions that significantly improved the faecal score compared to the other two groups. Other possible factors related to the risk of F4-ETEC PWD in SNP2+ piglets may include the pH of the stomach, variability in the exposure of F4 receptors in the intestinal epithelium, the gastrointestinal microbiome population of the host (as discussed by^53^) and potential susceptibility differences among the SNP2 genotype (heterozygous vs. homozygous susceptible; as indicated for MUC13 in^54^). Moreover, studies with other DNA marker-based tests have also reported associations between the marker (e.g. CHCF1, CHCF3, ALFA0072075) and F4-ETEC susceptibility^50,55,56^. Multiple markers are therefore considered as candidates, but the gene responsible for F4-ETEC susceptibility has not yet been elucidated, suggesting that further research is needed to understand the genetics of F4-ETEC susceptibility.

## Conclusion

The experiment confirmed that piglets fed with TOFA, YH or their combination had lower levels of faecal F4-ETEC shedding compared to the NC group, which was reflected in the reduced severity of diarrhoea, suggesting these dietary strategies improve the resilience of piglets towards F4-ETEC infection. F4-ETEC quantification and diarrhoea were significantly higher in SNP2+ piglets than in SNP2- piglets following an experimental F4-ac ETEC infection, enforcing previous observations of SNP2 as a suitable marker for F4ac-ETEC susceptibility.

## Methods

### Ethics declaration

The study was approved by the Dutch Central Authority for Scientific Procedures on Animals (project license AVD246002015279) and the Animal Care and Use Committee of Schothorst Feed Research (Lelystad, the Netherlands), and conducted at the research facility of SFR. The protocol of the experiment was carried out in compliance with the ARRIVE guidelines and in accordance with the Dutch law on animal experimentation, which complies with the European Directive 2010/63/EU on the protection of animals used for scientific purposes.

### Animals and selection criteria

Suckling piglets (75 Tempo x TN-70 piglets from 11 sows with a parity range from 1 to 5) were sampled for blood (1.2 ml) via jugular venepuncture at 21 days before weaning (approximately 9 days old). Blood was collected in EDTA tubes, and shipped with cool packs on the same day to Labo Dierlijke Genetica (Department of Veterinary and Life Sciences, Faculty of Veterinary Medicine, University of Gent, Merelbeke, Belgium) to determine the presence of SNP2 (ASGA0091537) by DNA analysis of whole blood. SNP2, identified by Goetstouwers et al.^24^ using the Porcine SNP60 BeadChip (Illumina), is positioned between HEG1 and MUC13, proximal to the Indel MUC13 marker (Indel of 68 bp in intron 2 of the MUC13 gene), and is suggested as a region related to an increased susceptibility to infections caused by enterotoxigenic *E. coli* F4^24,52^. The SNP2 marker test, a qPCR assay with dual-labelled probes, was developed and performed by Labo Dierlijke Genetica (Department of Veterinary and Life Sciences, Faculty of Veterinary Medicine, University of Gent, Merelbeke, Belgium). Based on the blood results, 25 SNP2-positive and 25 SNP2-negative piglets were selected at weaning, taking into account the weaning weight and sex of the piglets. SNP2-positive piglets were heterozygous susceptible (C/T), while SNP2-negative piglets were homozygous resistant (C/C).

At birth, piglets were ear tagged, and weighed, and their sex was determined and registered. No teeth clipping, tail docking or castration was performed. Litter size was standardized to 12–15 piglets per litter and cross-fostering was recorded. Litters were housed in farrowing pens with a size of 2.10 × 2.00 m and reared by the sow. Gilts and sows were vaccinated according to the manufacturer’s vaccination scheme with an inactivated vaccine against neonatal colibacillosis and *Clostridium* infections (SUISENG®, HIPRA, Amer, Girona, Spain). Piglets received commercial creep feed (ABZ Diervoeding, Leusden, the Netherlands) from approximately one week of age.

Piglets were selected for blood sampling purposes at 21 days before weaning based on the health status of the litter, average birth weight of the litter and sex (equivalent ratio). Piglets showing signs of injury or illness at the time of blood sampling or weaning were excluded from selection, as well as piglets that were cross-fostered or medically treated during the suckling period. Piglets were selected at weaning based on the following criteria: SNP2 detection in blood, sex (equivalent male to female ratio) and weaning weight. After being included in the study, piglets were excluded when a humane endpoint was met. Humane endpoints were defined as situations in which pigs were showing severe clinical symptoms, or moderate clinical symptoms during two observations without signs of recovery. In that case, they were humanely euthanized and sent for pathology to the GD (Deventer, the Netherlands). The trial started at 21 days pre-weaning, with blood sampling for SNP2 detection, and lasted for 43 days in total, of which 22 days post-weaning, with experimental treatments applied from weaning onwards.

A subset of 50 piglets was weaned (7.82 ± 0.146 kg) at 30 days of age (29.72 ± 0.128 days) and housed in 10 pens (2.00 × 1.00 m) in one climate-controlled room. Room temperature was automatically regulated by a climate computer following a temperature curve starting at 28 °C on the day of weaning to 23 °C at 22 days post-weaning. The room was ventilated using outdoor air. The humidity in the room was dependent on the outdoor humidity and ventilation rate. Artificial lights were provided from 7.00 to 17.00 h. Piglets had ad libitum access to feed and water. The pen floor was partly slatted and partly covered with a rubber mat. Each pen contained two feeding troughs with feeder space for 1 to 2 piglets per trough depending on pig body size, two drinking nipples, a metal chain with HDPE plastic bar that was alternated daily and a cotton rope.

### Experimental design and diets

A randomization process was performed to block piglets to pens based on weaning weight, SNP2 presence, and sex. The trial was setup as a complete randomized block design containing five treatments: Positive control (PC) with piglets fed with a negative control diet and provided with the antibiotic colistin via drinking water (Colisol® 250 000 I.E./ml; 10 ml product per 25 kg of body weight per day, Dopharma Research B.V., Raamsdonksveer, The Netherlands, REG NL 2182/UDD), that is known for the oral treatment of intestinal infections caused by *E. coli*, and particularly of PWD; Negative control (NC) with piglets fed with a negative control diet; Tall oil fatty acids (TOFA) with piglets fed with a negative control diet with the addition of 1.0 g per kg feed of TOFA containing 8.7% resin acids (PROGRES®; AB Vista, Marlborough, UK, see Supplementary Table 1, Additional File 1); Yeast hydrolysate (YH) with piglets fed with a negative control diet with the addition of 1.5 g per kg feed of YH derived from *Saccharomyces cerevisiae* (PROGUT® EXTRA; Hankkija Oy, Hyvinkää, Finland); and Combination (COM) with piglets fed with a negative control diet with the addition of 1.0 g TOFA and 1.5 g YH per kg feed. Each treatment group consisted of 10 piglets, divided over two pens, i.e. one replicate with lighter (on average 6.9 kg) and one replicate with heavier piglets (on average 8.7 kg) in terms of body weight. Treatments were given from weaning until the end of the experiment on day 22 post-weaning. In the PC group that received colistin throughout the 22 post-weaning days of the trial, the dosage was increased over time according to the piglets’ body weight development (10 ml product per 25 kg of body weight per day). In the first three days of the trial, all piglets were treated with colistin via drinking water to standardize and reduce the presence of ETEC in the intestine.

The experimental negative control diet was formulated to meet the requirements for all essential nutrients for piglets according to recommendations published by the Foundation Central Bureau for Livestock Feeding^57^. The diet was a barley, wheat, soybean meal, and maize-based diet. The diet composition and calculated nutrient contents are presented in Supplementary Table 2 of Additional File 1. The analysed nutrient contents of the diets are presented in Supplementary Table 3 of Additional File 1. The test products were added on top of the negative control diet to create the different treatments. Experimental diets were pelleted at a diameter of 3 mm in the specialised feed mill of ABZ Diervoeding (production facility in Leusden, the Netherlands). As method of blinding, numerical coding was used. Diet formulation, piglet selection and allocation, trial measurements and data analyses were performed by different personnel.

Moisture, crude protein, crude fat, crude fibre, and ash were determined in the diets using standard proximate analysis methods by the laboratory of Schothorst Feed Research (Lelystad, the Netherlands). The moisture determination was performed gravimetrically after oven-drying at 80 °C vacuum to a constant weight (NEN-ISO 6496:1999). Crude protein content was measured according to the Dumas principle (NEN-EN-ISO 16634–2:2016), crude fibre was determined by the filter technique (NEN-EN-ISO 6865:2001) and crude fat was determined after hydrolysis with hydrochloric acid under heating (NEN-ISO 6492:1999). Ash was measured gravimetrically after ashing the sample for 3 h at 550 °C (NEN-ISO 5984:2003). Proximate analysis of the experimental diets showed comparable contents of the Weende components. The analysed nutrient contents are presented in Supplementary Table 3 of Additional File 1.

The dietary concentration of resin acids was determined using gas chromatography (GC) and gas chromatography‒mass spectrometry (GC‒MS) methods by Oy Separation Research Ab (Turku, Finland). First, diet samples were ground to a homogeneous powder. Resin acids and free fatty acids were extracted using acetone and hexane, and analysed by GC and GC‒MS. Subsequently the concentration of TOFA in feed samples was calculated based on the analysed quantity of resin acids (see Supplementary Table 4, Additional File 1) and the known ratio of resin acids to free fatty acids in TOFA.

### Experimental challenge

On day 10 post-weaning, all piglets were orally inoculated via syringe with a 5 ml solution of 5.9 ± 2 × 10^9^ CFU of ET10, an enterotoxigenic *E. coli* strain (virotype: F4ac, STb, LT; serotype: O149:H10) isolated from a mild clinical case of PWD^55^. The whole-genome sequence is available at NCBI (ET10, BioProject ID: PRJNA770188). The inoculum was prepared by inoculating 1 L of HIB broth (OXOID HIB broth, Thermo Fisher, NL) with three colonies of ET10. After 24h at 37 °C, the inoculum was placed on ice, and a serial dilution was plated on Columbia blood agar (CBA plates, Tritium, NL). After inoculation of all piglets, another serial dilution was plated on Columbia blood agar plates to compare the bacterial concentration before and after inoculation.

### Measurements

Fresh faecal samples were collected from individual pigs after rectal stimulation at days 0, 8, 11, 12, 13, 14, 15, 18, 20 and 22 post-weaning. The faecal consistency of the samples was determined on an 8-point scale from severe water thin diarrhoea to hard, dry and lumpy faeces. The faecal consistency protocol can be found in Supplementary Table 5 of Additional File 1. To prevent dehydration, piglets with severe water thin diarrhoea were provided with a mixture of water plus electrolytes. The faecal dry matter of 69 selected faecal samples varying in faecal score was determined using an oven drying method (ISO 6496:1999) in which samples were weighed before and after drying at 80˚C for 18h. A faecal score of ≤ 4 was considered diarrhoea, while a faecal score of ≤ 4 in combination with F4-ETEC detected in the faecal sample on the same day was considered F4-ETEC diarrhoea.

After scoring the faecal consistency, the fresh faecal samples were placed on ice and taken to the laboratory. Approximately 2 g was taken from the samples at days 8, 11, 12, 13, 14, 15, 18, 20 and 22 post-weaning, their actual weight was recorded, and the samples were stored at –80 °C until shipment to BaseClear BV (Leiden, the Netherlands). DNA was extracted from each faecal sample and used to quantify faecal F4-ETEC using quantitative PCR (qPCR) targeting the inoculated F4-ETEC strain. DNA extraction, whole genome sequencing of the strains with the Illumina NovaSeq 6000 and assembly, qPCR assay screening, development and validation were performed at BaseClear BV (Leiden, the Netherlands) according to the methods provided in Additional File 2.

Individual body weight (BW, in kg) was recorded at the start of the experiment (weaning; day 0), as well as at days 8, 15, and 22 post-weaning. Average daily gain (ADG, g/piglet) was calculated for the separate periods (from day 0 to 8, day 8 to 15, and day 15 to 22 post-weaning) and for the total experimental period (from day 0 to 22 post-weaning). Feed allowance and refusals were recorded manually per pen (with 5 piglets per pen) at the same time points as the body weights were determined. Weights were determined using a platform scale (KERN FTB, KERN & Sohn GmbH, Balingen, Germany). The average daily feed intake (ADFI, g/piglet) and feed conversion ratio (FCR) were calculated on a pen basis for the separate periods and for the total experimental period.

Before F4-ETEC inoculation piglets were monitored daily for clinical symptoms (body condition, diarrhoea, behaviour, signs of dehydration), which increased to three times a day during the first five days post-inoculation and continued for two times a day until the end of the study. Additionally, rectal temperature was measured once a day during the first five days post-inoculation to monitor piglet health.

### Statistical analysis

No faecal sample, despite rectal stimulation, was obtained from one piglet on day 12 post-weaning, and it was thus designated as missing value for faecal consistency and ETEC excretion at this day. Data missing due to two piglets reaching a humane end point were handled as missing values in the dataset. The performance of these animals was taken into account until reaching the humane end point.

Each faecal sample was analysed in triplicate in the F4-ETEC qPCR, and the average cycle threshold (CT) was used to determine F4-ETEC concentrations. If one of the triplicates had an undetermined CT (≥ 40 cycles), then the average was taken of the CTs of the other two replicates. If two out of the triplicates had an undetermined CT, then the value of the replicate with determined CT (< 40 cycles) was used to quantify the F4-ETEC. The limit of detection (LOD) was set at 200 CFU F4-ETEC per gram of faeces. Samples with an undetermined CT (≥ 40 cycles) were given a value of 0.5 times the LOD. Finally, a log10 transformation was performed on the F4-ETEC concentrations before statistical analyses were performed.

The experimental data were analysed using GenStat® Version 23 for Windows™ (VSN International Ltd, Hemel Hempstead, UK). Missing values were estimated. The normality of the data was tested using Shapiro‒Wilk normality tests and residual plots. The piglet was the experimental unit for all response parameters. The main response parameters in the F4-ETEC challenge model are faecal F4-ETEC shedding (prevalence, duration and quantitative level) and F4-ETEC PWD (prevalence, duration and faecal consistency score). Treatment, genotype and their interaction were used as fixed effects unless stated otherwise. Piglet performance, duration of F4-ETEC diarrhoea, duration of faecal F4-ETEC shedding, and F4-ETEC concentration (on each day separately) were analysed using one-way analysis of variance (ANOVA) via the General Linear Model function. Faecal consistency and faecal F4-ETEC shedding over time were analysed in an ANOVA using treatment, day, and their interaction as fixed effects and replicate as a random effect, as well as using genotype, day, and their interaction as fixed effects and replicate as random effect. Faecal consistency at weaning (day 0 post-weaning) was used as a covariate in the model of faecal consistency over time. In case of a significant interaction with time, data were also analysed per day by ANOVA using treatment or genotype as a fixed effect and replicate as a random effect. Chi-square tests were used to analyse the prevalence of F4-ETEC diarrhoea and shedding. Significant differences are declared at *P* ≤ 0.05, with near significant trends at 0.05 < *P* ≤ 0.10. (Near) Significant fixed effects were further analysed by least significant differences (LSD, Fisher’s LSD method) to compare treatment means. Data are presented as absolute means, adjusted for covariates if any included. The *P*-value and standard error of the mean (SEM) are reported per response parameter.

## Supplementary Information

Supplementary Information 1.

Supplementary Information 2.

## Data Availability

The datasets generated and/or analysed during the current study are not publicly available due to their proprietary nature but are available from the corresponding author on reasonable request.
