# Author Correction: Effect of dietary tall oil fatty acids and hydrolysed yeast in SNP2-positive and SNP2-negative piglets challenged with F4 enterotoxigenic *Escherichia coli*

**DOI:** 10.1038/s41598-025-98620-w

**Published:** 2025-04-25

**Authors:** Anouschka Middelkoop, Hannele Kettunen, Xiaonan Guan, Juhani Vuorenmaa, Ramon Tichelaar, Michela Gambino, Martin Peter Rydal, Francesc Molist

**Affiliations:** 1 Schothorst Feed Research B.V., 8218 NA Lelystad, The Netherlands; 2 Hankkija Oy, 05800 Hyvinkää, Finland; 3 Department of Veterinary and Animal Sciences, Faculty of Health and Medical Sciences, University of Copenhagen, 1870 Frederiksberg C, Denmark

Correction to: *Scientific Reports* 10.1038/s41598-024-52586-3, published online 24 January 2024

In the original version of this Article, the project license was incorrect.

As a result, in the Methods section, under the subheading ‘Ethics declaration’:

“The study was approved by the Dutch Central Authority for Scientific Procedures on Animals (project license AVD104002016515)”

now reads:

“The study was approved by the Dutch Central Authority for Scientific Procedures on Animals (project license AVD246002015279)”

The original Article has been corrected.

