# Supplementary material for: Effect of dietary tall oil fatty acids and hydrolysed yeast in SNP2-positive and SNP2-negative piglets challenged with F4 enterotoxigenic Escherichia coli
Source: Sci Rep. 2024 Jan 24;14:2060. doi: 10.1038/s41598-024-52586-3 (PMC10808182; doi:10.1038/s41598-024-52586-3)
Supplement: Supplementary file 1 — Supplementary Information 1. [file 41598_2024_52586_MOESM1_ESM.docx]

# Supplementary Methods

**Effect of dietary tall oil fatty acids and hydrolysed yeast in SNP2-positive and SNP2-negative piglets challenged with F4 enterotoxigenic *Escherichia coli***

A. Middelkoop, H. Kettunen, X. Guan, J. Vuorenmaa, R. Tichelaar, M. Gambino, M.P. Rydal and F. Molist

*Scientific Reports*

**qPCR assay**

**DNA extraction**

Bacterial cells of the strain were propagated in peptone water for DNA isolation. Total DNA from the strain and from the stool samples were extracted with the ZymoBIOMICS DNA Miniprep Kit (Zymo Research Europe GmbH, Freiburg, Germany), following the manufacturer’s recommendations. Briefly, samples were added to the vial containing the ZR BeashingBead Lysis Tube beads (0.1 and 0.5 mm), and after adding 750 μL ZymoBIOMICS Lysis Solution, the vial was capped tightly and samples were mechanically disrupted using a Precellys 24 (Bertin Corp., Rockville, MD, USA) for 5 min s at 9000 rpm. DNA was eluted in 50 μL and stored at -20 °C for whole genome sequencing and for qPCR determination of copy numbers per gram of faecal sample.

**Whole Genome Sequencing of enterotoxigenic *E. coli* isolated from diarrheic pigs in central Netherlands**

Two complete genome sequences of *E. coli* strains isolated from pigs were selected as reference genomes (Shepard et al., 2012): Strain UMNK88, which is a F4 (K88) strain with serotype O149:H10 possessing the K88, LT, and STb genes, and lacking *stx*2e. The second was UMNF18 strain, which is an F18 strain with serotype O147:H4 possessing the F18, STa, and STb genes and the *stx*2e Shiga toxin gene. Serotypes were checked with the serotype finder of CGE <https://cge.cbs.dtu.dk/services/>. DNA extraction, whole genome sequencing of the strains with the Illumina NovaSeq 6000 and assembly were performed at BaseClear BV (Leiden, the Netherlands).

**Quantification of copy numbers of *E. coli* K88 F4 fimbriae-expressing gene**

qPCR assay screening, development and validation was performed at BaseClear BV (Leiden, The Netherlands). Whole-genome alignments of target and off-target genomes were performed *in silico* and yielded 20 optimal pairs of primers. Primer specificity for candidate primer pairs was confirmed using the Primer-BLAST tool . Blast hits with an identity score lower than 80% were regarded as negative, when taking the full length of the oligos into consideration (100% coverage). Primers for constructing the standard and for quantifying the ETEC phylotype were designed using this pipeline (Table S5).

Table S6. Primers used for detection for ETEC-F4 phylotype in the present study.

| Sample name | Sequence 5’-3’ |  |
| --- | --- | --- |
| For_EColiF4_136212 | GTTCTGTGAAGACGCAGCGT | Forward primer |
| Probe_EColiF4_136212 | GGAGATGTTTGGTAAGTACC | Probe |
| Rev_EColiF4_136212 | CGCCTCTTTCTGGATGAATAGTCTT | Reverse primer |

Standard curves were constructed using serial dilutions of plasmid DNA from a clone identified as ETEC K88-F4. Briefly, the sequence coding for the F4 fimbriae was used as template, blunt cloned into a pUC57-noBsaI vector, and linearized with Eco31I (BsaI, ThermoFisher Scientific, Landsmeer, The Netherlands). The restricted standard was loaded on a 1% agarose gel, aseptically excised and purified using the Zymo Gel DNA Recovery Kit (Zymo Research Europe GmbH, Freiburg, Germany).

A 6-zone gradient Real-time PCR assay was performed using a Veriti 96-Well Thermal Cycler (ThermoFisher Scientific, Landsmeer, The Netherlands) to determine the optimal annealing temperature of the designed primers. Optimal annealing temperature was 60°C, where the highest amplicon concentration and the lowest primer dimer formation were detected.

Efficiency of the assay was completed after determination of optimal annealing temperature. A qPCR test run of 10 serial dilutions of the ETEC K88-F4 standard was performed on an Applied Biosystems™ QuantStudio™ 5 Real-Time PCR System in a 384-wells format (ThermoFisher Scientific, Landsmeer, The Netherlands), using the following conditions: 95°C for 15 min; 40 cycles of 95°C for 15 s, 60°C for 1 min, and 95°C for 15 s; and a final step of 60°C for 1 min. Data generated from reactions with efficiencies between 90 and 110% were used for further analysis.
