# Supplementary material for: Effect of dietary tall oil fatty acids and hydrolysed yeast in SNP2-positive and SNP2-negative piglets challenged with F4 enterotoxigenic Escherichia coli
Source: Sci Rep. 2024 Jan 24;14:2060. doi: 10.1038/s41598-024-52586-3 (PMC10808182; doi:10.1038/s41598-024-52586-3)
Supplement: Supplementary file 2 — Supplementary Information 2. [file 41598_2024_52586_MOESM2_ESM.docx]

# Supplementary Tables and Figures

**Effect of dietary tall oil fatty acids and hydrolysed yeast in SNP2-positive and SNP2-negative piglets challenged with F4 enterotoxigenic *Escherichia coli***

A. Middelkoop, H. Kettunen, X. Guan, J. Vuorenmaa, R. Tichelaar, M. Gambino, M.P. Rydal and F. Molist

*Scientific Reports*

**Supplementary Table 1** Chemical composition of tall oil fatty acid (Progres^®^)

| Compound | Value |
| --- | --- |
| Free fatty acids, % |  |
| 18:2 - 9,12 Linoleic acid | 38.6 |
| 18:1 - 9 cis Oleic acid | 29.1 |
| 18:3 - 5,9,12 Pinolenic acid | 7.0 |
| 18:2 conjugated linoleic acid | 5.8 |
| 20:1 - 11 | 1.7 |
| 20:3 - 5,11,14 | 1.3 |
| 18:0 Stearic acid | 1.0 |
| Other free fatty acids | 4.8 |
| Free fatty acids, total | 89.3 |
| Resin acids, % |  |
| Abietic acid | 2.8 |
| Dehydroabietic acid | 1.3 |
| Pimaric acid | 1.0 |
| Palustric acid | 0.6 |
| Other resin acids | 2.5 |
| Resin acids, total | 8.2 |
| Unsaponifiables, % | 2.5 |
| Total | 100 |

# Supplementary Table 2 Diet composition and calculated nutrient values

|  | | Unit | Negative control diet |  |
| --- | --- | --- | --- | --- |
| Barley |  | % | 25.0 |  |
| Wheat |  | % | 20.0 |  |
| Soybean meal (<48% CP) | | % | 15.7 |  |
| Maize | | % | 15.0 |  |
| Whey protein delac (18% Ash) | | % | 5.0 |  |
| Soy protein concentrate (Soycomil) | | % | 3.0 |  |
| Maize gluten meal (60% CP) | | % | 3.0 |  |
| Molasses beet | | % | 2.5 |  |
| Soybean oil | | % | 2.0 |  |
| Sunflower meal (37% CP) | | % | 2.0 |  |
| Poultry fat |  | % | 2.0 |  |
| Limestone | | % | 1.0 |  |
| Water |  | % | 1.0 |  |
| Sodium bicarbonate |  | % | 0.80 |  |
| Monocalcium phosphate | | % | 0.43 |  |
| Vitamin/mineral premix^1^ |  | % | 0.60 |  |
| Salt | | % | 0.22 |  |
| Copper sulphate (99%) | | % | 0.05 |  |
| Choline-chloride (75%) | | % | 0.03 |  |
| Phytase | | % | 0.003 |  |
| *L*-Lysine HCL (79%) | | % | 0.41 |  |
| *L*-Threonine (98%) | | % | 0.09 |  |
| *L/DL*-Methionine (99%) | | % | 0.09 |  |
| *L*-Tryptophane (98%) |  | % | 0.03 |  |
| Moisture |  | g/kg | 123.6 |  |
| Ash |  | g/kg | 57.7 |  |
| Crude protein |  | g/kg | 201.0 |  |
| Crude fibre |  | g/kg | 27.8 |  |
| Starch |  | g/kg | 346.5 |  |
| Crude fat (Acid hydrolysis) |  | g/kg | 65.3 |  |
| Net Energy (NE) |  | MJ/kg | 9.7 |  |
| SID Lys |  | g/kg | 11.5 |  |
| SID Lys/ NE | | ratio | 1.32 |  |
| SID Met/ SID Lys | | ratio | 0.32 |  |
| SID M+C/ SID Lys | | ratio | 0.57 |  |
| SID Thr/ SID Lys | | ratio | 0.63 |  |
| SID Val/ SID Lys | | ratio | 0.71 |  |
| SID Trp/ SID Lys | | ratio | 0.20 |  |
| SID His/ SID Lys | | ratio | 0.38 |  |
| SID Arg/ SID Lys |  | ratio | 0.91 |  |
| SID Ile/ SID Lys | | ratio | 0.64 |  |
| SID Leu/ SID Lys | | ratio | 1.30 |  |
| SID Phe/ SID Lys |  | ratio | 0.76 |  |
| Available Ca |  | g/kg | 7.50 |  |
| Digestible P |  | g/kg | 3.76 |  |
| Zn |  | mg/kg | 91.92 |  |
| Cu |  | mg/kg | 149.94 |  |
| NSP |  | g/kg | 139.76 |  |

^1^ Vitamin/mineral premix was added to provide the following nutrients per kg of diet: Vitamin A: 6,500 IU; Vitamin B_1_: 1.2 mg; Vitamin B_2_: 5.6 mg; Vitamin B_3_ (Niacin): 38 mg; Vitamin B_5_ (Calcium D-panthotenate) 14.1 mg, Vitamin B_6_ (Pantothenic acid): 1.2 mg; Vitamin B_7_ (Biotin): 0.12 mg; Vitamin B_9_ (Folic acid): 0.4 mg; Vitamin B_12_: 28 mcg; Vitamin D_3_: 1,600 IU; Vitamin E: 60 mg; Vitamin K_3_: 1.35 mg; I: 0.55 mg; Se: 0.35 mg; Cu: 15 mg; Fe: 115.8 mg; Mn: 45.4 mg; Zn: 62.1 mg.

**Supplementary Table 3** Analysed dietary nutrient contents for the experimental diets, in g/kg

| **Diet code** | **Description** | **Moisture** | **Ash** | **CP** | **CFat** | **CF** |
| --- | --- | --- | --- | --- | --- | --- |
| A | NC | 102.8 | 54.0 | 198.3 | 60.1 | 30.2 |
| B | TOFA | 100.3 | 55.4 | 197.2 | 63.0 | 30.4 |
| C | YH | 100.4 | 55.7 | 196.8 | 62.5 | 29.8 |
| D | COM | 105.6 | 56.0 | 197.0 | 60.2 | 28.8 |

**Supplementary Table 4** Analysed resin acids (RA) and added and calculated tall oil fatty acid (TOFA) contents for the experimental diets, in g/kg

| **Diet code** | **Description** | **TOFA added** | **RA**  **analysed** | **TOFA calculated** |
| --- | --- | --- | --- | --- |
| A | NC | 0 | 0 | 0 |
| B | TOFA | 1.0 | 0.108 | 1.24 |
| C | YH | 0 | 0 | 0 |
| D | COM | 1.0 | 0.108 | 1.24 |

**Supplementary Table 5** Faecal consistency scoring protocol for weaned piglets

| Score | Description |
| --- | --- |
| 2 | Water thin faeces. Flows through slatted floor. |
| 3 | Liquid faeces. Flows through slatted floor. |
| 4 | Loose faeces, like custard. |
| 5 | Smooth, shapeless pile of faeces. |
| 6 | Mushy faeces without structure, like peanut butter.  Sticks to glove when picked up. |
| 7 | Firm and shaped faeces without structure (smooth). |
| 8 | Firm and shaped faeces with structure (cracks on the surface).  Able to be picked up as a whole. |
| 9 | Hard, dry and lumpy faeces.  After being picked up, the glove is not or hardly dirty. Falls apart after shaking. |

**Supplementary Table 6** The interaction effect between treatment and genotype on faecal F4-ETEC shedding in log_10_ CFU / g faeces at each of the measurement days of the experimental period in piglets of diverse genetic background fed with different dietary strategies. F4-ETEC inoculation took place on day 10 post-weaning

| Treatment | PC^1^ | NC | TOFA | YH | COM | SEM | *P*-value |
| --- | --- | --- | --- | --- | --- | --- | --- |
| SNP2+ piglets | | | | | | | |
| Day 8 | 2.00 | 2.00 | 2.00 | 2.00 | 2.00 | - | - |
| Day 11 | 5.56 | 6.56 | 6.74 | 7.89 | 6.87 | 0.477 | 0.30 |
| Day 12 | 4.63 | 7.63 | 7.32 | 7.56 | 7.23 | 0.658 | 0.55 |
| Day 13 | 3.12 | 7.69 | 5.70 | 7.77 | 7.61 | 0.703 | 0.48 |
| Day 14 | 2.31 | 7.52 | 5.62 | 6.86 | 6.04 | 0.809 | 0.97 |
| Day 15 | 2.37 | 7.73 | 5.00 | 7.43 | 5.76 | 0.869 | 0.68 |
| Day 18 | 2.22 | 3.97 | 2.90 | 3.68 | 3.21 | 0.479 | 1.00 |
| Day 20 | 2.00 | 3.03 | 2.62 | 2.02 | 2.00 | 0.343 | 0.64 |
| Day 22 | 2.00 | 2.93 | 1.97 | 2.04 | 2.74 | 0.504 | 0.69 |
| SNP2- piglets | | | | | | | |
| Day 8 | 2.00 | 2.00 | 2.00 | 2.00 | 2.00 |  |  |
| Day 11 | 4.91 | 7.07 | 6.42 | 6.27 | 6.22 |  |  |
| Day 12 | 3.18 | 5.66 | 3.90 | 4.68 | 4.12 |  |  |
| Day 13 | 2.00 | 6.14 | 4.44 | 4.47 | 4.99 |  |  |
| Day 14 | 2.00 | 6.11 | 4.40 | 5.57 | 5.04 |  |  |
| Day 15 | 2.40 | 6.57 | 4.39 | 4.95 | 5.19 |  |  |
| Day 18 | 2.00 | 3.83 | 2.76 | 3.15 | 2.95 |  |  |
| Day 20 | 2.00 | 3.46 | 2.32 | 2.23 | 2.72 |  |  |
| Day 22 | 2.00 | 3.34 | 2.67 | 3.04 | 2.39 |  |  |

Abbreviations: PC = control diet + colistin via drinking water; NC = control diet; TOFA = 1.0 g tall oil fatty acids per kg feed; YH = 1.5 g yeast hydrolysate from *Saccharomyces cerevisiae* per kg feed; COM = 1.0 g TOFA and 1.5 g YH per kg feed

**Supplementary Table 7** The interaction effect of genotype and experimental treatment on the performance of weaned piglets challenged with F4-ETEC^1^

| Description | PC | NC | TOFA | YH | COM | SEM | *P*-value |
| --- | --- | --- | --- | --- | --- | --- | --- |
| SNP2+ piglets |  |  |  |  |  |  |  |
| Body weight, kg |  |  |  |  |  |  |  |
| D 0 | 7.87 | 7.98 | 8.07 | 7.62 | 7.64 | 0.508 | 0.90 |
| D 8 | 8.65 | 9.23 | 9.15 | 8.69 | 8.55 | 0.581 | 0.57 |
| D 15 | 11.81 | 9.53 | 11.47 | 11.11 | 10.20 | 0.631 | 0.35 |
| D 22 | 15.70 | 12.98 | 15.47 | 14.64 | 13.76 | 0.866 | 0.64 |
| Average daily gain, g/piglet | | | | | |  |  |
| D 0 – 8 | 98 | 156 | 135 | 134 | 114 | 38.6 | 0.32 |
| D 8 – 15 | 452 | 145 | 334 | 343 | 235 | 45.6 | 0.17 |
| D 15 – 22 | 556 | 492 | 571 | 505 | 509 | 55.3 | 1.00 |
| D 0 – 22 | 356 | 249 | 337 | 319 | 278 | 31.4 | 0.57 |
| SNP2- piglets |  |  |  |  |  |  |  |
| Body weight, kg |  |  |  |  |  |  |  |
| D 0 | 7.90 | 7.63 | 7.51 | 7.88 | 7.91 |  |  |
| D 8 | 8.46 | 8.93 | 8.87 | 9.20 | 9.89 |  |  |
| D 15 | 11.62 | 11.57 | 11.81 | 12.25 | 12.06 |  |  |
| D 22 | 15.49 | 15.06 | 15.81 | 15.86 | 15.59 |  |  |
| Average daily gain, g/piglet | | | | | |  |  |
| D 0 – 8 | 70 | 163 | 170 | 165 | 247 |  |  |
| D 8 – 15 | 453 | 377 | 422 | 433 | 310 |  |  |
| D 15 – 22 | 552 | 497 | 572 | 516 | 505 |  |  |
| D 0 – 22 | 345 | 338 | 378 | 362 | 349 |  |  |

^1^F4ac-ETEC challenge on day 10 post-weaning

Abbreviations: PC = control diet + colistin via drinking water; NC = control diet; TOFA = 1.0 g tall oil fatty acids per kg feed; YH = 1.5 g yeast hydrolysate from *Saccharomyces cerevisiae* per kg feed; COM = 1.0 g TOFA and 1.5 g YH per kg feed

**Supplementary Fig. 1** The effect of day on faecal consistency of weaned piglets upon F4-ETEC challenge on day 10 post-weaning. The grey-coloured background indicates the pre-inoculation period

**Supplementary Fig. 2** The effect of day on faecal F4-ETEC shedding of weaned piglets upon F4-ETEC challenge on day 10 post-weaning. The grey-coloured background indicates the pre-inoculation period

**Supplementary Fig. 3** The effect of treatment and genotype on the duration of F4-ETEC shedding in weaned piglets upon F4-ETEC inoculation on day 10 post-weaning. PC, control diet + colistin via drinking water; NC = control diet; TOFA = 1.0 g tall oil fatty acids per kg feed; YH = 1.5 g yeast hydrolysate from Saccharomyces cerevisiae per kg feed; COM = 1.0 g TOFA and 1.5 g YH per kg feed
